# Spontaneous regression of an interhemispheric arachnoid cyst: illustrative case

**DOI:** 10.1007/s00381-024-06464-y

**Published:** 2024-05-18

**Authors:** Angelica M. Fuentes, John A. Jane

**Affiliations:** 1 Department of Neurosurgery, University of Virginia, Charlottesville, VA USA; 2 Department of Neurosurgery, Virginia Tech Carilion School of Medicine, Carilion Clinic, Roanoke, VA USA

**Keywords:** Arachnoid cyst, Interhemispheric cyst, Spontaneous regression, Pediatric

## Abstract

**Background:**

Intracranial arachnoid cysts are benign collections of cerebrospinal fluid that are often asymptomatic and discovered incidentally. An interhemispheric location of these lesions is rare, with only a few such cases reported in the literature. Though spontaneous regression of arachnoid cysts has been described in other locations, to date this phenomenon has not been reported in interhemispheric fissure cysts.

**Observations:**

In this report, we describe a patient with a large, multiloculated interhemispheric arachnoid cyst diagnosed on prenatal ultrasound. She did not exhibit neurologic deficits or signs of increased intracranial pressure and was observed with serial imaging. After several years of observation, imaging revealed spontaneous and progressive decrease in the cyst size.

**Lessons:**

We illustrate a case of regression of an interhemispheric arachnoid cyst in a pediatric patient. To our knowledge, this is the first reported case of spontaneous shrinkage of an arachnoid cyst in this location. Although the current presentation is rare, this reporting adds to the current understanding of natural history of arachnoid cysts and provides an example of radiographical improvement without intervention of a cyst located within the interhemispheric fissure.

## Introduction

Intracranial arachnoid cysts are congenital, benign collections of cerebrospinal fluid surrounded by an arachnoid membrane that are often asymptomatic [1, 2]. Interhemispheric location of these cysts is uncommon, with only a few cases reported in the literature [3–5]. Interestingly, several reports have detailed cases of cyst shrinkage or disappearance without intervention in locations other than the interhemispheric region. The literature for interhemispheric cysts in the pediatric population focuses primarily on their surgical management [4, 6–9].

Here, we report a case of a large interhemispheric cyst that spontaneously decreased in size after conservative management. To the authors’ knowledge, this is the first description of spontaneous reduction of an interhemispheric arachnoid cyst.

## Illustrative Case

**Fig. 1:**
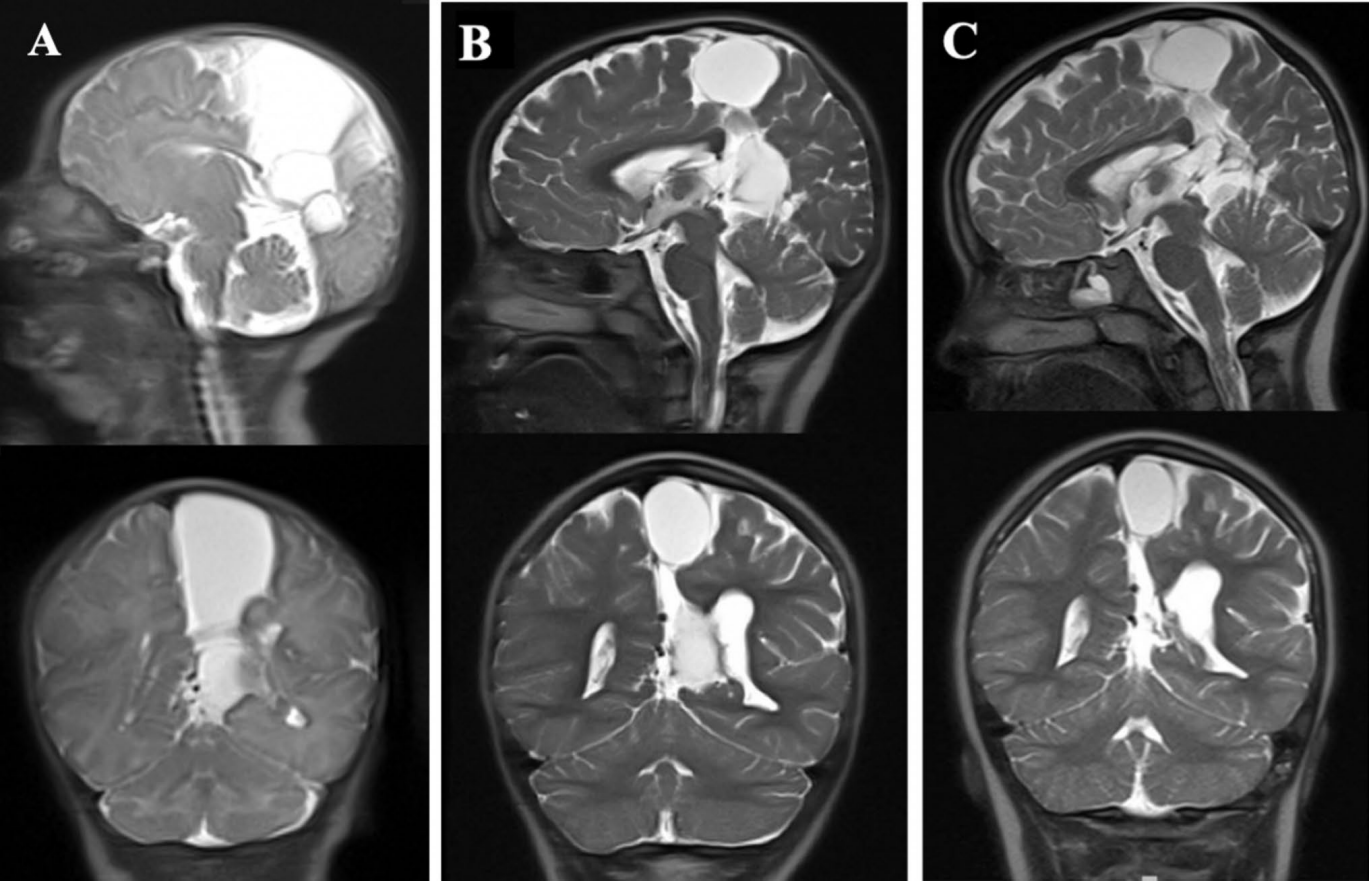
**A** Sagittal (upper) and coronal (lower) brain fast sequence magnetic resonance imaging (MRI) on day 2 of life, demonstrating a large cluster of extra-axial cystic structures at the medial aspect of the left parietal lobe extending inferior to the quadrigeminal cistern with mass effect on the adjacent brain and left lateral ventricle, as well as hypoplastic posterior corpus callosum. **B** Sagittal (upper) and coronal (lower) brain fast sequence MRI at 6 years of age, showing decrease in size of the cyst. **C** Sagittal (upper) and coronal (lower) brain fast sequence MRI at 8 years of age, which showed further decrease in size in the cyst

This female patient had an unremarkable medical history aside from a multiloculated interhemispheric arachnoid cyst that was discovered during routine prenatal ultrasound. Subsequent ultrasounds in utero demonstrated stability in size of the cystic mass. She was born at 38 weeks via C-section due to arrest of descent. Delivery was also notable for prolonged rupture of membranes (22 h) with chorioamnionitis, for which her mother was treated with ampicillin and gentamicin. Head circumference at birth was in the 75th percentile, and the baby’s fontanelle was soft and compressible. Fast sequence magnetic resonance imaging (MRI) was obtained on day 2 of life to establish a baseline, which demonstrated a large cluster of extra-axial cystic structures within the interhemispheric space, with associated hypoplastic posterior corpus callosum (Fig. 1A). No intervention was planned at that time as the baby did not show any signs of increased intracranial pressure (ICP) or neurological deficit from the cyst. She was discharged on day 4 of life, and outpatient neurosurgical follow-up was arranged. Repeat cranial imaging was obtained at her first follow-up at age 3 weeks, which showed unchanged size of the interhemispheric cyst and stable hypoplasia of the splenium of the corpus collosum. Head circumference at this time was near the 50th percentile. Imaging at follow-up over the next 5 years revealed stability of cyst size. Head circumference over that time did increase to approximately the 80–90th percentile, which also remained stable thereafter. She did not exhibit motor deficits, experience headaches, or have symptoms of increased ICP such as papilledema throughout this time. Brain MRI at her follow-up at age 6 years showed significant reduction in the cyst size compared to prior (Fig. 1B). Further decrease in cyst size was revealed on MRI at her 8-year follow-up visit (Fig. 1C), after which follow-up was planned on an as-needed basis. She continued to do well clinically and did not exhibit any neurologic deficit.

## Discussion

### Observations

As interhemispheric arachnoid cysts are rare lesions, information regarding their natural course and optimal management is limited. It is estimated that interhemispheric location of arachnoid cysts comprises approximately 5% of all cases [10]. One retrospective study of arachnoid cyst prevalence in children found that of 309 incidentally detected cysts, only 4 were located in the interhemispheric region [11]. In children, these lesions are typically concomitant with developmental anomalies of midline structures, such as partial or complete agenesis of the corpus callosum [3, 6–9]. Our patient demonstrated dysplastic appearance of the splenium of the corpus callosum, aligning with this association. In spite of the large dimensions these lesions can attain, clinical manifestations may be subtle or absent. With the increased utilization of ultrasound prenatally, more of these lesions have been diagnosed prior to birth [12, 13], as was the case in our patient.

Arachnoid cysts associated with neurological symptoms or increased ICP can be managed surgically with craniotomy for cyst resection, endoscopic fenestration, or cystoperitoneal shunting. There have been several prior case series on the surgical management of interhemispheric cysts using such techniques [2, 6, 7, 9, 10]. This literature demonstrates that when encountered, interhemispheric cysts are often treated operatively.

Regression or disappearance of arachnoid cysts has been reported in prior literature. There have been several cited instances of cyst disappearance following head injury, in which cysts found in association with subdural or epidural hematomas following trauma decrease in size as the hematoma subsides [14–16]. One proposed mechanism of this phenomenon is that cranial trauma may cause rupture of the outer membrane of the cyst, thus leading to cyst fluid drainage into the subarachnoid space [17]. Thereby, cysts found incidentally on imaging done after trauma are found to decrease in size on subsequent imaging. Other types of intracranial irritation aside from trauma, such as meningitis, have also been associated with cyst disappearance [18]. In the absence of trauma or other notable irritating event, some have suggested that a communication between the cyst and subarachnoid space can be created by sudden, transient increases in ICP during Valsalva maneuver [19]. Numerous cases of seemingly spontaneous regression of arachnoid cysts have been discussed in the literature in both children and adults [20–26]. The most common locations of these reported disappearing arachnoid cysts are the middle cranial fossa, posterior cranial fossa, temporal/temporo-frontal region, suprasellar region, and prepontine region [19]. To date, there has been no cited case of spontaneously regressing interhemispheric cyst.

In the current case, we describe a pediatric patient with a multiloculated arachnoid cyst diagnosed in utero that was followed with serial imaging for several years and subsequently demonstrated spontaneous shrinkage. She did not have cranial trauma nor other intracranial irritation or pathology that preceded cyst regression. Of note, during the period of observation of the cyst, the patient experienced a fall from her tricycle without consequent loss of consciousness or neurological change. However, this occurred after radiological evidence of decrease in cyst size. As such, this fall likely was not the inciting event that led to cyst regression.

In cases of asymptomatic arachnoid cysts, most opt for conservative treatment with observation and serial imaging. Reports of shrinkage of these lesions are clinically relevant when counseling patients and families on the potential outcomes of arachnoid cysts. The currently reported case is the first cited description of such a shrinkage of an arachnoid cyst located within the interhemispheric fissure, adding to the collective knowledge of possible clinical course of a cyst in this specific location.

## Data Availability

No datasets were generated or analysed during the current study.
